# Universal Poisson Statistics of mRNAs with Complex Decay Pathways

**DOI:** 10.1016/j.bpj.2015.12.001

**Published:** 2015-12-30

**Authors:** Mukund Thattai

**Affiliations:** 1 Simons Centre for the Study of Living Machines, National Centre for Biological Sciences, Tata Institute of Fundamental Research, Bangalore, India

## Abstract

Messenger RNA (mRNA) dynamics in single cells are often modeled as a memoryless birth-death process with a constant probability per unit time that an mRNA molecule is synthesized or degraded. This predicts a Poisson steady-state distribution of mRNA number, in close agreement with experiments. This is surprising, since mRNA decay is known to be a complex process. The paradox is resolved by realizing that the Poisson steady state generalizes to arbitrary mRNA lifetime distributions. A mapping between mRNA dynamics and queueing theory highlights an identifiability problem: a measured Poisson steady state is consistent with a large variety of microscopic models. Here, I provide a rigorous and intuitive explanation for the universality of the Poisson steady state. I show that the mRNA birth-death process and its complex decay variants all take the form of the familiar Poisson law of rare events, under a nonlinear rescaling of time. As a corollary, not only steady-states but also transients are Poisson distributed. Deviations from the Poisson form occur only under two conditions, promoter fluctuations leading to transcriptional bursts or nonindependent degradation of mRNA molecules. These results place severe limits on the power of single-cell experiments to probe microscopic mechanisms, and they highlight the need for single-molecule measurements.

## Main Text

The small volume of living cells and the small number of many important biological molecules forces us to adopt a discrete description of biochemical reactions. Randomness or stochasticity arises as an immediate consequence: when we move from concentrations to molecule numbers, we move from reaction rates to reaction probabilities per unit time. As a result, for any molecule of interest, isogenic cells in identical conditions will have a broad distribution of molecule number. The variance of the distribution—the extent of deviation from the deterministic value—is particularly large for macromolecules such as messenger RNA (mRNA) and proteins, which are synthesized rarely and exist in small numbers.

The influence of stochasticity on gene expression, often referred to as intrinsic noise in the transcription of mRNA and the translation of proteins, has been well studied both theoretically and experimentally (1, 2, 3). Simple birth-death models of transcription and decay predict that steady-state mRNA numbers, *m*, should follow a Poisson distribution whose variance, $\sigma_{m}^{2}$, is equal to its mean, $\mu_{m}$ (4). Measurements of mRNA expression at an inducible promoter in the bacterium *Escherichia coli* showed that variance did scale with the mean, though the Fano factor, $\sigma_{m}^{2}/\mu_{m}$, was ∼4 rather than unity (5). A comprehensive analysis of mRNA numbers for over a thousand *E. coli* promoters found that variance scaled with the mean over two orders of magnitude, and that the median Fano factor, $\sigma_{m}^{2}/\mu_{m}$, was ∼1.6 (6), close to the Poisson expectation. Inferred mRNA fluctuations based on protein abundance measurements in the yeast *Saccharomyces cerevisiae* suggested that mRNAs were Poisson distributed (7). Finally, direct single-RNA counting experiments in *S. cerevisiae* showed that mRNA distributions of many housekeeping genes were of the Poisson form (8).

In these analyses, the term Poisson is used in three related but distinct ways. First, a Poisson process is a memoryless process in which events occur with a constant probability per unit time, implying an exponential distribution of interevent intervals. Second, a Poisson distribution is the distribution of the number of events generated by a Poisson process in a fixed time interval, a result also known as the law of rare events. Third, the steady-state distribution of the number of molecules when birth and death are Poisson processes coincidentally also takes the mathematical form of the Poisson distribution, often called the Poisson steady state.

It has been suggested that measured mRNA and protein distributions can be used to probe the underlying microscopic synthesis and decay dynamics (9). Taking the Poisson steady state as a null model, it is assumed that any deviations can be attributed to “non-Poissonian mRNA production or degradation” (6). Conversely, it is assumed that Poisson scaling “reflects fluctuations in mRNA levels that arise from the random birth and death of individual mRNA molecules” (7). Both claims are too strong. Transcriptional bursts or more complicated promoter dynamics (10, 11) indeed generate non-Poisson steady-state mRNA distributions. However, complex models of decay such as senescence generate Poisson scaling (12, 13). These results hint that there might be a deeper reason why observed mRNA distributions for *E. coli* (5, 6) and *S. cerevisiae* (7, 8) are close to Poisson, though mRNA decay is known to be a regulated multistep process in both bacteria and eukaryotes (14, 15, 16).

Here, I give a proof of the universality of the Poisson steady state for mRNAs with Poisson synthesis dynamics but arbitrarily complex decay pathways. This follows from a fundamental one-to-one correspondence between these dynamics and the law of rare events, under a nonlinear rescaling of time. I emphasize the identifiability problem: a large class of distinct microscopic models have Poisson steady states and therefore cannot be distinguished by single-cell measurements. Finally, I characterize the precise conditions under which we expect deviations from the Poisson steady state. This discussion is self-contained, but throughout the text I highlight connections with results from queueing theory (see Gross et al. (17) and Kleinrock (18) for a description of queueing notation), which can be used as a starting point for more complex derivations.

We start with the usual birth-death process (the M/M/∞ queue) where ∅ represents the empty set, *M* represents mRNA, and *m* is the number of mRNA molecules at some time:

(1) $$\emptyset \overset{\alpha }{\to }M\overset{\gamma }{\to }\emptyset .$$

The stochastic chemical-kinetic system in Eq. 1 has a Poisson steady state with mean 〈m〉=α〈τ〉, where 〈τ〉=1/γ is the mean mRNA lifetime under exponential decay (4):

(2) $$P\left(m\right)=\frac{{\langle m\rangle }^{m}}{m!}\;e^{-\langle m\rangle }.$$

We now extend this to the case where mRNA synthesis is an inhomogeneous time-dependent Poisson process, and decay is a multistep, potentially branching and looping process through a variety of intermediate states $M^{\prime }$, $M^{\prime \prime }$, …. Here, *m* represents the total number of mRNA molecules across all such states. The decay reaction propensities could be constant, or could themselves be drawn from time-dependent statistical distributions. Schematically,

(3) $$\begin{array}{ccccccc} \emptyset & \to & M & \to & \cdots & \to & M^{\prime }\\ \; & \;\alpha \left(t\right) & ↑ & \left\{\gamma \left(t\right)\right\}\; & ↓ & \; & ↓\\ \; & \; & M^{\prime \prime } & \leftarrow & \cdots & \to & \emptyset \end{array}.$$

It is a surprising and powerful result that the transient mRNA distribution of the complex system in Eq. 3 has precisely the Poisson form of Eq. 2, where 〈m〉 is now the (possibly time-dependent) mean mRNA number (19, 20). If a steady state does exist, then 〈m〉=α〈τ〉, where 〈τ〉 is the mean mRNA lifetime under any stationary model of decay. Here, I attempt to convey the fundamental mathematical origins of the Poisson distribution within this diverse class of models.

**Figure 1:**
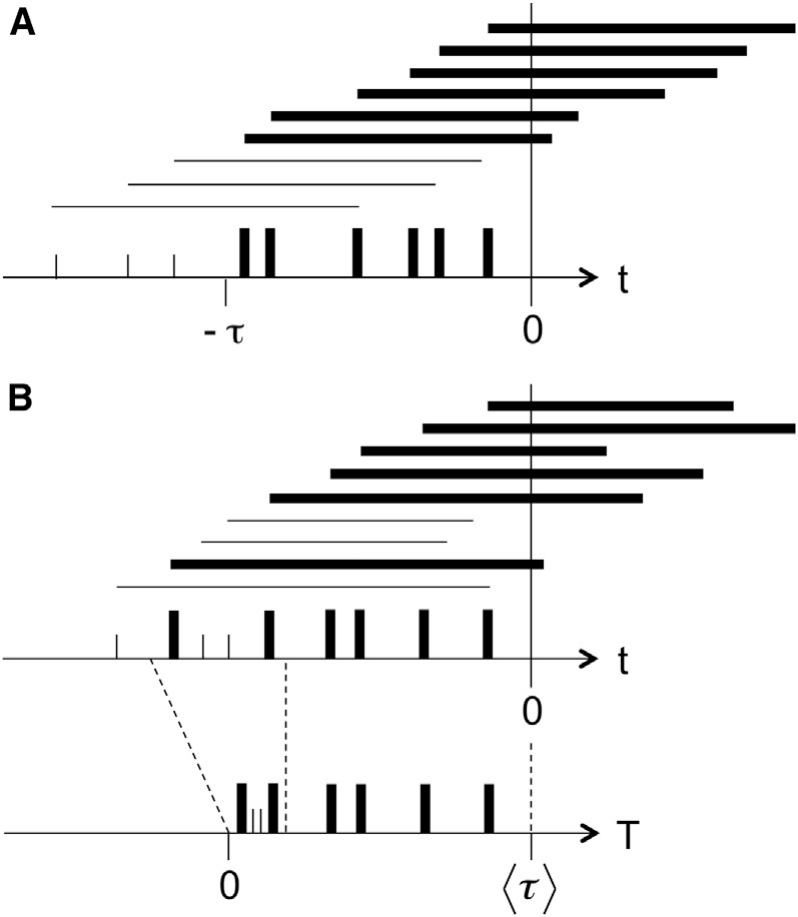
Synthesis and decay of mRNA. We sample mRNA molecules at time *t* = 0. Vertical ticks show mRNA synthesis events. Horizontal lines show the persistence and decay of single mRNA molecules. We keep track of synthesis events for molecules that survive at the sampling time (*bold, tall ticks*). Those that have decayed are ignored (*short ticks*). (*A*) The scenario where all mRNA molecules have the same lifetime, τ, is equivalent to having a constant rate of synthesis in the interval (−τ,0). (*B*) The scenario where mRNA molecules have a distribution of two lifetimes (also shown in Fig. 2, *right*). At the sampling time, all new mRNAs survive, but only long-lived old mRNAs survive. The effective synthesis rate thins out as we move to the past. This can be compensated for by a nonlinear change of variables to a new time variable, *T*, squeezing the time axis. This restores an effective constant rate of synthesis in an interval of width 〈τ〉, the mean mRNA lifetime.

Consider an idealized system in which every mRNA molecule has precisely the same lifetime, τ≡〈τ〉, (Fig. 1 *A*, the M/D/∞ queue). At any sampling time *t* = 0, the only mRNA molecules present will be those created in the time interval (−τ,0). This is the same as asking for the number of synthesis events of a Poisson process with rate α in a time interval of width τ. The result, by the law of rare events interpretation, is the Poisson distribution of Eq. 2. But what if the mRNA molecules have a distribution of lifetimes R(τ) (which can be obtained by a first-passage-time analysis of the decay pathways in Eq. 3)? In this case, the molecules present at the sampling time could have been created arbitrarily far back in time (Fig. 1 *B*), and there is no obvious way to invoke a law of rare events interpretation.

To proceed, consider Eq. 3 with constant propensities of synthesis and decay (Fig. 1 *B*; the M/G/∞ queue), and consider the probability of survival of an individual mRNA molecule for some time interval τ since its synthesis:

(4) $$S\left(\tau \right)=\int_{\tau }^{\infty }R\left(\tau^{\prime }\right)d\tau^{\prime }.$$

An mRNA molecule created at time –τ has a chance S(τ) of surviving to the sampling time *t* = 0. It is only these survivors we need to consider; all the molecules that have already decayed can effectively be ignored. This is the same as looking at the result of an inhomogeneous Poisson process with a time-dependent synthesis rate, αS(τ), and ignoring decay altogether. The effective synthesis rate thins out the farther back in time we go. Integrating into the past, this generates a Poisson distribution with mean

(5) $$\langle m\rangle =\int_{0}^{\infty }\alpha S\left(\tau \right)d\tau .$$

**Figure 2:**
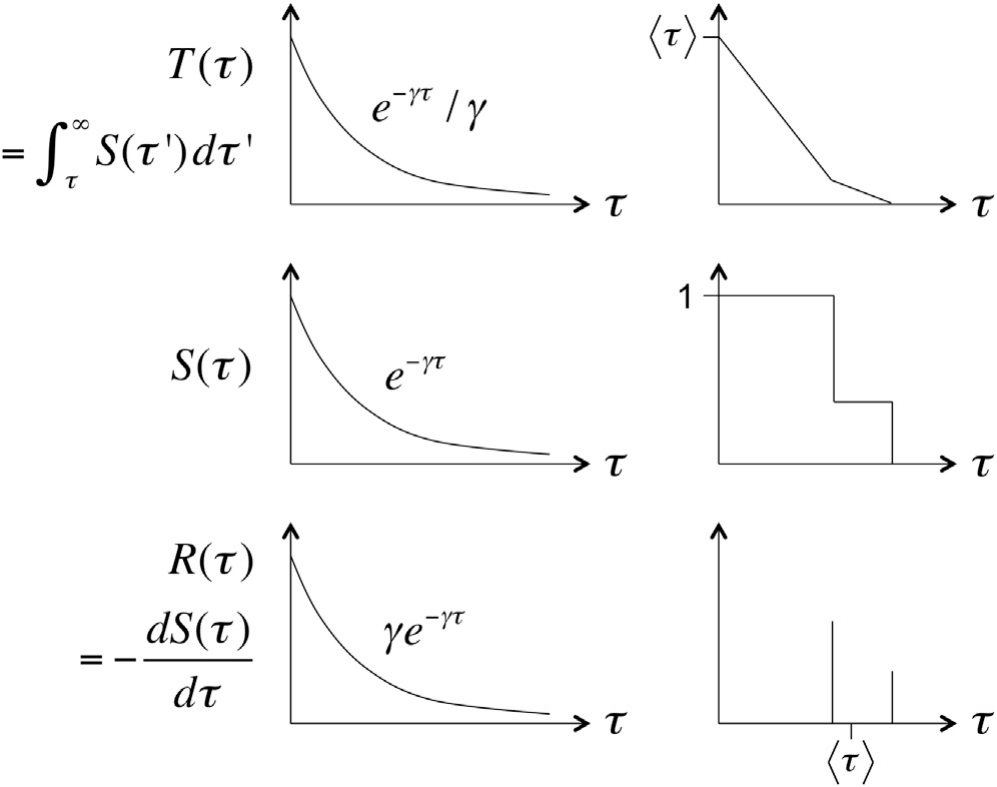
From lifetime distributions to rescaled time. (*Lower*) The lifetime distribution R(τ)=−dS(τ)/dτ. (*Middle*) The survival probability, S(τ). (*Upper*) The rescaled time variable, $T\left(\tau \right)=\int S\left(\tau^{\prime }\right)d\tau^{\prime }$. Integration by parts relates the functions R(τ) and T(τ) through Eq. 7. (*Left*) The standard birth-death process with an exponential lifetime distribution. (*Right*) A system in which each molecule can randomly have one of two possible lifetimes, represented as delta functions, corresponding to the dynamics shown in Fig. 1*B*. Note that τ measures time going into the past.

The key observation is that we could equivalently rescale time by the change of variables, |dT|=|S(τ)dτ|, so that synthesis again becomes a Poisson process with constant rate α. In effect, the thinning out of past synthesis events is compensated for by nonlinearly squeezing the time axis (Fig. 1 *B*). It only remains to fix what happens to the limits of the integral in Eq. 5 (Fig. 2). We define

(6) $$T\left(\tau \right)=\int_{\tau }^{\infty }S\left(\tau^{\prime }\right)d\tau^{\prime }$$

so that T(∞)=0, and through integration by parts,

(7) $$\begin{array}{c} T\left(0\right)={S\left(\tau \right)\tau |}_{0}^{\infty }-\int_{0}^{\infty }\frac{dS\left(\tau \right)}{d\tau }\tau d\tau \\ =\int_{0}^{\infty }R\left(\tau \right)\tau d\tau \equiv \langle \tau \rangle , \end{array}$$

where the boundary term goes to zero by a version of Markov’s inequality, assuming only that R(τ) has a finite mean (21). The variable *T* maps the infinite past to 0 and the sampling time to 〈τ〉 (Fig. 1 *B*). Therefore,

(8) $$\int_{0}^{\infty }\alpha S\left(\tau \right)d\tau =\int_{0}^{\langle \tau \rangle }\alpha dT=\alpha \langle \tau \rangle =\langle m\rangle ,$$

and we recover exactly a law of rare events picture in which synthesis is a Poisson process with constant rate α in a time interval of width 〈τ〉, precisely as in Eq. 2.

In queueing theory, the rightmost equality of Eq. 8 is known as Little’s law and relates various mean values (17, 18). We are more concerned with the leftmost equality, which rescales the time variable τ to a new time variable, T, and contains information about the entire distribution. The benefit of this point of view is that we can now extend the model to more complex situations. If we can find a rescaled time in which a law of rare events interpretation is valid, the distribution must be Poisson.

For example, suppose we had started with zero mRNA molecules and turned on synthesis at time $-\tau_{0}$. Then, at the sampling time *t* = 0, we would still have a Poisson distribution such that

(9) $$\begin{array}{c} T\left(\tau \right)=\int_{\tau }^{\tau_{0}}S\left(\tau^{\prime }\right)d\tau^{\prime }\\ \int_{0}^{\tau_{0}}\alpha S\left(\tau \right)d\tau =\int_{0}^{T\left(0\right)}\alpha dT=\alpha T\left(0\right)=\langle m\rangle , \end{array}$$

even though the system had not yet reached steady state.

We next consider more complicated cases with time-dependent rates. We assume that all functions of time are shifted so that the sampling time is *t* = 0. If the synthesis propensity is time-dependent but decay propensities are constant (the $M_{t}\text{/}G\text{/}\infty$ queue (19)), we have

(10) $$\int_{0}^{\infty }\alpha \left(\tau \right)S\left(\tau \right)d\tau =\int_{0}^{T\left(0\right)}\alpha \left(T\right)dT=\langle m\rangle ,$$

which is an inhomogeneous Poisson process in the rescaled time interval, whose mean depends on the sampling time. For the classic birth-death process with time-dependent synthesis and decay rates (the $M_{t}\text{/}M_{t}\text{/}\infty$ queue (20)), we could first rescale time to make the decay rate constant. This would then be a special case of the inhomogeneous Poisson process in Eq. 10, and we recover a Poisson distribution with time-dependent mean.

Finally, we consider the full-blown version of Eq. 3 with time-dependent synthesis and decay (the $M_{t}\text{/}Ph_{t}\text{/}\infty$ or $M_{t}\text{/}G_{t}\text{/}\infty$ queues (20)). All mRNAs synthesized at time *t* would have the same lifetime distribution, $R_{t}\left(\tau \right)$, and their probability of survival would be

(11) $$S_{t}\left(\tau \right)=\int_{\tau }^{\infty }R_{t}\left(\tau^{\prime }\right)d\tau^{\prime }.$$

We now examine $S_{-\tau }\left(\tau \right)$, the probability that an mRNA synthesized at time –τ survives to the sampling time. Confusingly, $S_{-\tau }\left(\tau \right)$ need not decrease monotonically into the past, though it must go to zero in the infinite past. For example, an epoch of long-lived mRNAs followed by an epoch of short-lived mRNAs would mean older mRNAs had a greater chance of surviving to the present. Nevertheless, the rescaled time variable is a well-defined monotonic function with a Poisson interpretation:

(12) $$\begin{array}{c} T\left(\tau \right)=\int_{\tau }^{\infty }S_{-\tau^{\prime }}\left(\tau^{\prime }\right)d\tau^{\prime }\\ T\left(0\right)=-\int_{0}^{\infty }\frac{dS_{-\tau }\left(\tau \right)}{d\tau }\tau d\tau \\ \int_{0}^{\infty }\alpha \left(\tau \right)S_{-\tau }\left(\tau \right)d\tau =\int_{0}^{T\left(0\right)}\alpha \left(T\right)dT=\langle m\rangle . \end{array}$$

Note that $-dS_{-\tau }\left(\tau \right)/d\tau \neq R_{-\tau }\left(\tau \right)$ (the subscript in Eq. 11 is *t*, not τ) so T(0) does not have a simple interpretation as the mean of some lifetime distribution.

Having discussed the large number of circumstances where the Poisson distribution arises, I will mention where it does not. Clearly, if the synthesis process is not Poisson there is no time rescaling that can make it Poisson. This is the situation when the promoter has fluctuating internal dynamics (the $Ph_{t}\text{/}Ph_{t}\text{/}\infty$ queue (20)), or the transcripts arrive in bursts (the $M^{X}\text{/}G\text{/}\infty$ queue (22)). More interestingly, if mRNAs interact so that their decay is correlated, for example, by saturating the degradation machinery (e.g., the M/G/k queue (17)), it is in general not possible to define a state-independent lifetime distribution or rescaled time, and mRNA numbers will not be Poisson distributed (however, see Grima (13) for some exceptions).

Ultimately, Poisson-distributed mRNA numbers are expected to be ubiquitous. The underlying argument is transparent, requiring no master equations or recursion relations, and it generalizes to complex decay pathways and transients. For all models with Poisson-distributed transients or steady states, the entire distribution is parameterized by the time-dependent mean, 〈m〉(t), which is the solution of an ordinary differential equation. As such, it is impossible to distinguish microscopic models using single-cell-resolved measurements, so true single-molecule measurements are warranted. For non-Poisson cases, measurement of carefully chosen means and variances can be used to probe microscopic details (22).

Does the squeezed time have any physiological correlate, beyond its mathematical utility? Here, we find an intriguing connection to psychophysics: the mRNA survival probability is a past temporal discounting function, so the rescaled variable *T* is subjective time (23): it is the cell’s perception of the flow of recent events, as sampled by the mRNAs present at some instant. This has implications for the notion of memory in living systems.
